# Hierarchies in eukaryotic genome organization: Insights from polymer theory and simulations

**DOI:** 10.1186/2046-1682-4-8

**Published:** 2011-04-15

**Authors:** Balaji VS Iyer, Martin Kenward, Gaurav Arya

**Affiliations:** 1 Department of NanoEngineering, University of California San Diego, 9500 Gilman Drive, La Jolla, CA 92093-0448, USA

## Abstract

Eukaryotic genomes possess an elaborate and dynamic higher-order structure within the limiting confines of the cell nucleus. Knowledge of the physical principles and the molecular machinery that govern the 3D organization of this structure and its regulation are key to understanding the relationship between genome structure and function. Elegant microscopy and chromosome conformation capture techniques supported by analysis based on polymer models are important steps in this direction. Here, we review results from these efforts and provide some additional insights that elucidate the relationship between structure and function at different hierarchical levels of genome organization.

## Introduction

The term "genome" refers to the complete linear DNA sequence containing all of the hereditary material possessed by an organism. One of the goals of the human genome project was to determine the sequence of the 3 billion base pairs that constitute human DNA. Despite the wealth of information this *tour-de-force *of scientific enterprise has generated, it is increasingly becoming clear that the cellular function of the human genome is not merely determined by the linear ordering of its DNA base pairs. In fact, many of the functional aspects of the genome are governed by its three dimensional (3D) structure, which involves meters long DNA packaged into the limiting space of a micrometer sized cell nucleus.

**Figure 1:**
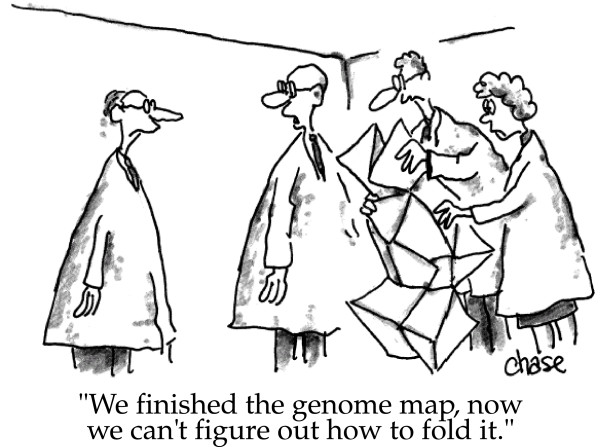
**Illustration of the Genome Folding Problem**. Illustration of the important question on genome organization. (Adapted with permission from cartoonist John Chase-http://www.chasetoons.com)

The DNA, thus packaged, occupies a significant portion of the nucleus volume while cellular factors that read, copy, modify, and maintain the genome, occupy the remaining. Ultimately, sophisticated patterns in cellular function arise due to a coupling between the accessibility of genetic information in the packaged DNA, and the organization and activity of cellular factors within the cell nucleus. For instance, nuclear processes like transcription, translation, repair and recombination do not occur ubiquitously in the nucleus, but are spatially compartmentalized in transcription, replication and recombination factories [1-3]. Clearly, how the 3D organization of the genome modulates these nuclear processes and how the nuclear processes in turn modify genome structure are important questions in modern cell biology. A critical step in addressing these questions requires a fundamental understanding of the genome 3D structure and the physical principles governing its organization, as articulated concisely yet powerfully in the cartoon of Figure 1.

**Figure 2:**
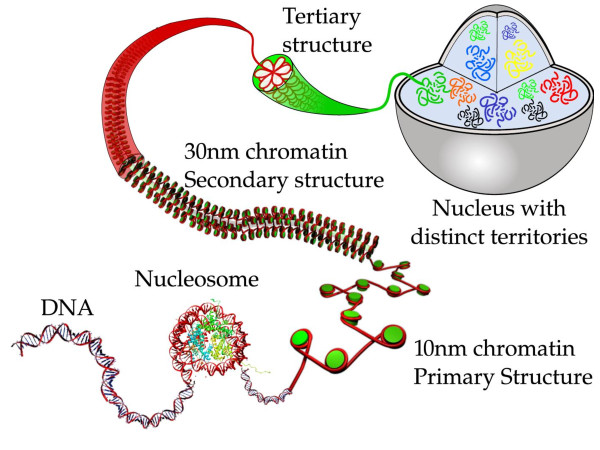
**Hierarchies of Genome Organization**. The hierarchical process by which eukaryotic double-stranded DNA (two meters long, in the case of humans) is packaged within the confines of a micrometers-sized cell. As shown schematically in the figure, this process encompasses *three *main organization levels classified as primary, secondary and tertiary [115,116].

Increasingly, ideas from polymer theory and simulations coupled with state-of-the-art microscopy and chromosome conformation capture techniques are being used to determine the 3D structure of the genome and the physical principles governing its folding. In this article, we present an overview of the key aspects and insights gained from these studies at the different hierarchical levels of organization shown in Figure 2. We begin with a discussion of mesoscale models and simulation methods used to decipher the secondary structure of the genome, the folded chromatin fiber, on the scale of 1-10 kbp. Next, we discuss coarse-grained models and simulations of the genome tertiary structure. At the tertiary structure level genome compaction varies all the way from ~50 to 100,000 fold, therefore, we have chosen to divide it into two sub-structures: tertiary-*α *structure at the gene locus level (10-2000 kbp) and tertiary-*β *structure at the chromosomal level (1-200 Mbp).

## Primary and Secondary Structure: Chromatin Organization

The classic image of double stranded DNA (dsDNA) is that of a naked double helix. However, in eukaryotes, dsDNA seldom occurs in its naked form. Instead, it is packaged through a ubiquitous hierarchical process involving specialized proteins called histones. This packaging serves two purposes. First, it compacts the DNA allowing it to fit into the confines of the cell nucleus, and second, it controls the accessibility of DNA to cellular machinery for transcription, regulation, repair and recombination [4].

The *primary *structure of the eukaryotic genome consists of DNA wrapping ~1.7 times around histone octamers comprising of two copies of the four histone proteins H2A, H2B, H3, and H4 [4-7]. The combined histone octamer-DNA complex is called the nucleosome. This nucleosomal organization of DNA is considered to be the primary determinant to accessibility of genetic information. Although the atomic structure of the nucleosome has been resolved through X-ray crystallography [6,7], the dynamics of nucleosomes remains far from fully understood. However, theoretical modeling and simulations are beginning to provide new insights into nucleosomal dynamics by answering questions related to: (1) how histone post-translational modifications and histone variants affect nucleosome structure and intra/inter-nucleosome interactions and (2) how nucleosomes undergo spontaneous conformation transitions between fully- and partially-wrapped states.

Recently, we have [8] employed all-atom molecular dynamics (MD) simulations and molecular docking to examine the structure of the H4 histone tail and its interactions with the acidic patch of nucleosomes. The H4 tail was found to exhibit a propensity for an *α*-helical conformation within a stretch of six residues encompassing the lysine 16 (K16) residue. Interestingly, this *α*-helical region interacted very strongly with the acidic patch; K16, in particular, mediated strong electrostatic interactions with the negatively-charged residues of the acidic patch. Acetylation of K16 diminished these binding interactions, suggesting a plausible mechanism by which post-translational acetylation of K16 could trigger chromatin unfolding [9]. To examine nucleosomal dynamics beyond the time scales accessible to all-atom simulations, Sharma et al. [10] and Voltz et al. [11] have developed coarse-grained models of the nucleosome. The former has helped in the identification of important histone residues, termed "cold sites", that maintain the stability of the histone octamer and the latter has been used to compute long wavelength fluctuations of the nucleosome. Some effort has also been devoted to elucidating the energy barriers and the kinetic rate constants associated with the accessibility of DNA in the nucleosome [12] and explaining specific features of force-induced nucleosome unraveling observed in single-molecule experiments [12-14].

Beyond the single nucleosome level, contiguous nucleosomes separated by short sections of naked DNA called linker DNAs yield the classic beads-on-a-string structure depicted in Figure 2. The *secondary *structure of chromatin involves folding of this beads-on-a-string motif into a ~30-nm thick fiber called chromatin. While, *in vivo *imaging of cell nuclei has yielded little information on the secondary structure of chromatin, and has even brought into question the very existence of a 30-nm fiber [15,16], electron microscopy of isolated nucleosomal arrays have been successful in imaging the transition from 10-nm beads-on-a-string structures to 30-nm condensed structures with increasing salt concentrations [15,17]. Regardless of the gaps in our knowledge of the secondary structure of chromatin *in vivo*, this structure is expected to dictate the accessibility of DNA sequences for interactions with nuclear machinery and is also likely to play a role in the recruitment of histone modifying and remodeling factors to specific regions of the genome. Local packing of nucleosomes within the chromatin fiber could also potentially affect its interactions with distant portions of the fiber or other chromatin fibers through processes like interdigitation [18].

Despite decades of research, the internal structure of the 30-nm chromatin fiber remains controversial. This controversy arises because of two main reasons: (1) *in vivo *chromatin is too "messy" to be visualized with even the most advanced microscopy techniques [15] and (2) *in vitro *reconstituted nucleosome arrays are too large and flexible to be crystallized, and too compact at physiological conditions for their linkers to be fully resolved through microscopy. These limitations have led researchers to adopt indirect ways of deducing the internal structure of chromatin, using chemical cross-linking and single-molecule pulling techniques, with varying degrees of success. Based on the data, two types of models for the chromatin structure have been proposed that differ mainly in the location and configuration of the linkers. In the one-start solenoid model, linkers exhibit a strongly bent configuration and reside at the fiber interior [17,19,20]. In the two-start helix, linkers exhibit a straight or gently bent configuration, and they could reside at the periphery of the fiber in one version of the model [21] or inside the fiber (close to its axis) in a zigzag manner in another version [22-24].

Computational modeling has played an integral role in providing new insights into chromatin architecture. The main challenge in modeling chromatin is the vast degrees of freedom possessed by even small segments of the fiber (e.g., an array of 50 nucleosomes contains >1 million atoms). Hence, all-atom approaches demand prohibitive amounts of computational resources to converge to equilibrium structures. Further, the larger and the more flexible the system, the less adequate a description by one single equilibrium conformation because of the large variation in possible structures around thermal equilibrium. In other words, the systems become "fuzzy" and can only be described in terms of statistical averages. The advantage of this situation is that atomic-detail resolution may easily be abandoned for vast gains in computational speed in a coarse-grained model. To this end, a major focus of computational modeling has been on developing lower-resolution models or "force fields" of nucleosome arrays that still account for the energetic interactions and constraints between the different components of the arrays. A large effort in computational modeling also lies in developing efficient procedures for "sampling" low-energy conformations of the nucleosome array subject to appropriate force fields.

**Figure 3:**
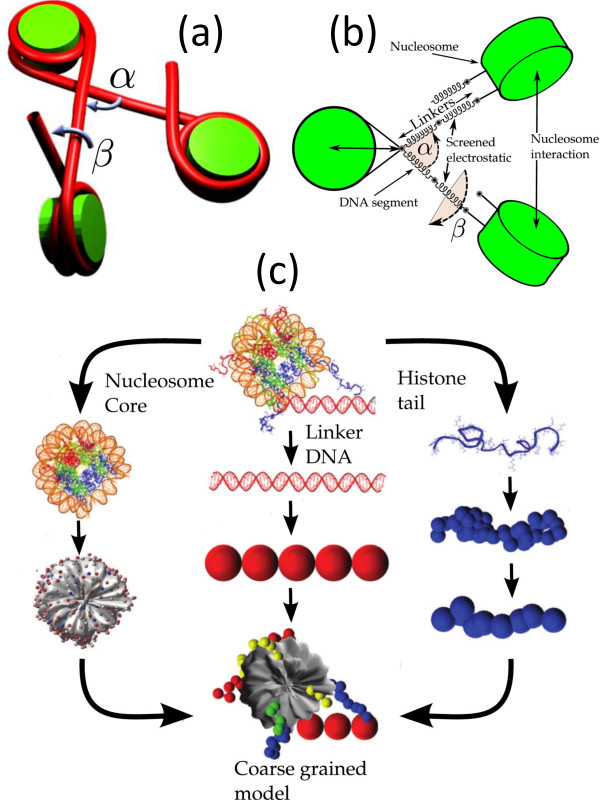
**Chromatin Modeling**. Coarse grained models of 10 nm chromatin fiber with different level of details. (a) The simple two angle model has two parameters, the angles *α *and *β *[25]. (b) The detailed two angle model includes energy terms for stretching and bending of the bead-chain with an additional energy term for the relative twist angle between adjacent beads to account for the twisting rigidity of DNA [37]. (c) Representation of the detailed mesoscale model of Arya et al [44,45].

A range of computational models that include widely differing amounts of detail have been developed. The simplest of these models are the so-called "two-angle" models [25,26], where the nucleosome arrays are modeled using two physical parameters: the entry-exit angle *α *of the linker DNA and the angle *β *describing the relative rotation between consecutive nucleosomes, the latter being determined by linker length *L *(Figure 3a). Woodcock et al. [25] illustrated how *α *and *β *(or *L*) play a key role in dictating the 3D structure of the resulting nucleosome array. At fixed *α*, variations in *L *lead to stark changes in chromatin structure and variations in the nucleosome packing ratios. The authors also showed that small variations in *L *introduce sharp bends in the fiber axis similar to those observed in cryo-electron microscopy of moderately-folded arrays, possibly due to overwrapping/underwrapping of nucleosomal DNA or displacement of nucleosomes along the DNA. Two-angle models with consideration for the excluded volume of nucleosomes have been extremely useful in providing sterically permissible conformations of nucleosomal arrays for specified constraints on nucleosome packing ratio, fiber diameter and linker length [27-31].

The E2A model is an extension of the two angle model [25] that also accounts for the cylindrical shape of the nucleosomes [32]. Simulations based on the E2A model are ideally suited for studying long nucleosomal arrays as they overcome the difficulty of lack of knowledge of actual interaction potentials between nucleosomes by using model parameters obtained from experimental data. In particular, the E2A model has been used for studying the effects of variability in linker lengths and variability in linker histone occupancy [32]. In combination with high-resolution light microscopy experiments these simulations promise a way to capture local chromatin structure in the range of 10 *- *40 nm resolution [33].

The next class of models, in addition to including the features of the two-angle model, include the mechanics and electrostatics of the linker DNAs along with a simple treatment of inter-nucleosome interactions [34-36]. The linker DNA is treated as a discretized wormlike chain, possessing energy terms for stretching and bending of the bead-chain with an additional energy term for the relative twist angle between adjacent beads to account for the twisting rigidity of DNA [37] (Figure 3b). The linker beads are also assigned suitable effective charges that interact with each other via the Debye-Hückel potential [38]. The inter-nucleosome interactions have thus far been treated as hard spheres, interacting with a hard-core attraction [34] or as cylinders interacting with Gay-Berne [35,36] and Zewdie potentials [36]. The nucleosome array conformations and packing ratios obtained using Monte Carlo simulations [35,36] based on these models generally resemble those obtained by electron microscopy for moderately folded chromatin. These models [34] have also provided valuable insights into the stretching behavior of chromatin and yielded first estimates of the strength of internucleosome interactions by matching simulated force-extension curves to those obtained experimentally [39]. Importantly, the models have revealed the sensitivity of the chromatin structure to the internucleosome interaction strength and linker lengths [36]. Furthermore, it was observed that nucleosome arrays exhibit a zigzag structure without the linker histone and a more solenoidal structure for linker histone-bound arrays [40].

Very detailed models of local interactions within oligonucleosomes have been developed by Schlick and coworkers [41-49] (Figure 3c). The earliest model [41,42] treated the nucleosome as a rigid cylinder (along with a small protrusion to represent the H3 histone tail) with hundreds of "pseudo" charges scattered uniformly on the surface. The magnitude of the charges were optimized to reproduce as closely as possible the electric field in the vicinity of the nucleosome. This approach allowed one to account for the salt-dependence in the inter-nucleosome interactions. The model was later refined by employing an irregular-shaped representation of the nucleosome [43] that was based on a more recent nucleosome crystal structure with all histone tails fully resolved [50]. The model reproduced the experimentally observed [15,17] compaction of the arrays with increasing salt concentration and indicated that the arrays maintain a zigzag morphology under monovalent salt conditions. Further, the simulations demonstrated that reduced electrostatic repulsion between the linkers is the main mechanism responsible for the folding of arrays at high salt. These models are also being used to study the dynamics of chromatin arrays, especially under different kinds of forces including torsional stresses [51].

Recently, this model was further improved by accounting for histone tail flexibility [44], linker histone binding [47], and effects of divalent ions [47]. The tails were treated as coarse-grained bead-chains, where each bead represented five amino acid residues. The stretching, bending, and the electrostatic terms in the bead-chain were parametrized using an iterative procedure. The linker histone was coarse-grained as three charged beads rigidly bound at the nucleosome dyad with the magnitude of the charges optimized to reproduce the electric field of the atomic linker histone. Divalent ions were treated phenomenologically in terms of their effect on flexibility and electrostatic screening of the linker DNAs. A configurational-bias Monte Carlo approach was used to sample the tail configurations and translation, rotation and pivot moves were used to sample the global array configurations [46]. This model helped elucidate the role of each histone tail, the linker histone and physiological salt condition in chromatin folding [45,49]. Specifically, the H4 tails were found to mediate the strongest internucleosome interactions, the H3 tails mediated strong internucleosome interactions and screened electrostatic repulsion between the entering/exiting linkers, and the H2A and H2B tails mediated inter-fiber interactions [45]. The linker histones constricted the linker entry/exit angle to bring alternate nucleosomes together. Divalent ions were also found to facilitate tight packing of nucleosomes by allowing a fraction of the linkers to bend and by strongly screening the linker repulsion at the fiber axis. Moreover, the model, in conjunction with sophisticated cross-linking experiments, confirmed the existence of a heteromorphic fiber containing both zigzag and solenoid conformations in the presence of additional divalent cations and linker histones [49]. This model has also recently been used to reproduce the linker length dependence in the observed chromatin structures [48].

In summary, mesoscale models at varying levels of sophistication such as those discussed above are proving to be valuable tools for examining chromatin structure. The choice of which model to use is dictated by the amount of detail required and the amount of computational resources available. For instance, the detailed models accounting for histone tail flexibility and nucleosome geometry may provide the most accurate representation of short nucleosome arrays. For long arrays containing hundreds of nucleosomes, these models rapidly become computationally intractable and the intermediate-resolution models like the E2A model [32] become more suitable.

## Tertiary-*α *Structure: Gene Locus Organization

In the previous section, we discussed the secondary structure of chromatin from a static perspective. In reality, the chromatin fiber within the cell nucleus is present in a dynamic state [52]-it is flexible over lengths much larger than the fiber diameter [53] and it is constantly being subjected to various kinds of remodeling activities, including histone modifications [54-56], sliding and depletion of nucleosomes [57-64], and incorporation of histone variants [65]. There is strong evidence from light microscopy studies indicating that at the gene locus level the chromatin fiber is organized into loops [66]. Studies on several multigene clusters conceive such loops as instrumental in bringing together distant enhancer and promoter regions crucial for gene activation, regulation and recombination [67-69]. Although the detailed mechanism of looping is not fully understood, there is no question that the "intrinsic" bending rigidity of the chromatin fiber dictates to a considerable degree the loop size-dependent statistical probability of two distant regions of the fiber coming into close proximity to form a loop.

Emerging evidence suggests that the relationship between flexibility and looping probability may be utilized by cells for gene regulation. Specifically, the idea that modulation of flexibility through remodeling processes like acetylation of histones alters the probability of interactions between a remote enhancer and cognate gene by means of looping has been successfully applied to explain gene regulation of the *Hoxd *gene cluster and the *β*-globin locus [70]. Strong support for this idea also comes from recent experiments in the Murre lab that show large-scale conformational changes in the *IgH*-locus during B-Cell development accompanying genome-wide and locus-specific histone modifications and nucleosome depletion events [66,71,72]. Thus, it seems that chromatin remodeling events, apart from modulating the local structure of chromatin and DNA accessibility, could lead to changes in the higher-order folding of chromatin through its effects on macroscopic properties of the fiber such as its flexibility.

So far, little effort has been devoted to investigating the intrinsic flexibility of chromatin and the associated looping probability as a function of loop size; especially how the flexibility correlates with external conditions like monovalent/divalent salt concentration and system parameters like nucleosome repeat length, DNA wrapping angle, histone variants, histone modifications and presence/absence of linker histones. Chromatin flexibility as characterized by its persistence length, *L_p_*, seems to exhibit large variations, depending on the experimental method used for its determination and the conditions and type of chromatin investigated. For example, single-molecule pulling of nucleosome arrays at low salt [39] and *in vivo *looping driven cross-linking/recombination assays [53,73] measure *L_p _*as low as 30-50 nm while analysis of fluorescent markers in the genome of erythrocytes measure *L_p _*of 100-200 nm [74,75]. However, it is important to review these results in light of a recent fundamental study [76] indicating that standard definitions of persistence length as used in these studies may not describe the local intrinsic flexibility of chromatin.

Aumann et al. [77] recently examined *L_p _*of nucleosome arrays using Monte Carlo simulations of a mesoscale model of chromatin. The persistence length was obtained from the decay in the correlation of the tangent vector representing the local fiber axis. It was found that *L_p _*decreased strongly with increasing nucleosome repeat length and increasing entry/exit angle, and that binding of the linker histone led to an increase in *L_p_*, consistent with experimental observations of linker histone deficient and inclusive chromatin. A comparison between the magnitude of the bending and elastic rigidity suggested that chromatin is much easier to bend than stretch, leading to the interesting hypothesis that it may be easier for the cells to pack chromatin via tight loops rather than by linear compression of the fiber.

**Figure 4:**
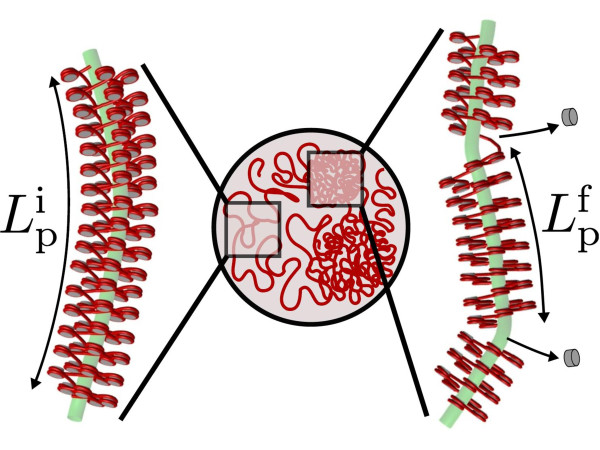
**Nucleosome Depletion**. Schematic of nucleosomal depletion associated persistence length modification and consequent local conformation change in the cell nucleus.  and  refer to the initial and final persistence length, respectively.

As discussed earlier, chromatin exists in a highly dynamic state within the cell nucleus. In fact, histone octamers are constantly being dissolved and rebound with the average genome-wide nucleosome occupancies being less than 75% [32]. Heermann and coworkers [78,79] have recently examined the effects of such depletion events on the persistence length and conformation of nucleosome arrays (Figure 4). They employed an extended two-angle (E2A) model [25,32], which allows examination of very long nucleosome arrays containing *>*1000 nucleosomes. An adaptation of the E2A model with experimental distribution of nucleosome repeat lengths yields a quantitative estimate for persistence length modification. Recent Monte Carlo (MC) simulations based on this adaptation indicate a decrease of *L_p _*from an initial value of 280 nm to 140 nm with ≈ 20% increase in the nucleosome skip probability [78]. This leads to sharp bends in the fiber allowing for formation of loops in the kilo base pair range [79], an important feature of genome organization visualized in experiments [69].

We have recently begun to examine the mechanisms behind the conformational collapse in the *IgH *- locus observed by Murre and coworkers during B-cell development [66]. We hypothesize that chromatin remodeling events (nucleosome depletion and histone modifications [71,72]) introduce flexible "hinges" within the chromatin fiber causing it to collapse (Figure 4). By treating the chromatin fiber as a worm-like chain (WLC) with fixed contour length *L_c _*and variable persistence length *L_p_*, we showed that the compaction, as characterized by the ratio of the final to initial mean square end-to-end distances, is given by the ratio of the final to initial persistence length in the ideal chain limit (see Appendix for complete derivation). Using the initial and final *L_p _*observed by Heermann and coworkers [78], this ratio becomes 1/2, indicating that the gene locus compacts by ≈30%. Interestingly, 3D distance measurements in the *IgH*-locus during progressive stages of B-cell development show a similar ~30% decrease in locus dimensions in response to remodeling events [66]. To examine the functional implications of this collapse, we used a simple Flory type argument to show that the collapse leads to an 8-fold increase in the number of binary interactions *n_b _*within the gene locus. Assuming that the frequency of promoter-enhancer (P-E) interactions is proportional to *n_b_*, we conclude that the conformational collapse facilitates transcriptional regulation and/or recombination by allowing for higher probability of P-E interactions. Thus, the chromatin remodeling induced changes in the persistence length offers one possible mechanism for governing the conformational state of the gene locus and consequent modification of the functional state of the cell (Figure 4).

## Tertiary-*β *Structure: Chromosomal Organization

Interestingly, the condensed higher-order structures of chromatin, namely chromosomes, were observed as distinct entities during mitosis as early as the 19^th ^century, much before the primary and secondary structures of chromatin were known to exist. However, disappearance of this condensed structure during interphase and the underlying chromatin organization remained a mystery till the late 20^th ^century [80]. In the beginning two different models were proposed for the interphase chromatin organization: (1) random organization akin to a bowl of spaghetti without any apparent structure and (2) organization in territories that later condense during mitosis to form distinct chromosomes [81]. The earliest clues to deciding between the two models came from experiments shining a laser light onto a specific volume of the cell nucleus and observing the effects of the consequent damage on replication [81]. It was found that only a few chromosomes were affected by the laser light, indicating existence of distinct chromosome territories within the cell nucleus. This conjectural evidence has now been confirmed beyond doubt by advances in imaging techniques like fluorescent in situ hybridization (FISH) [82-84] and chromosome painting [82,83].

While there is concrete evidence for the existence of territories, the internal architecture of chromatin within chromosome territories, the interaction between territories, and the organizational principles governing their formation remain poorly understood [85]. Results from MC simulations of confined polymer chains suggest that territory formation could arise from simple, non-specific entropic forces and from segregation of long chains attempting to conserve their topological state while undergoing confined Brownian motion [86,87]. Increasingly, insights from such coarse-grained polymer models coupled with experiments are being used to examine the internal architecture of chromosome territories.

Two predominant themes recur during discussions of higher-order structures of interphase chromosomes: (1) formation of loop structures and (2) confined fractal organization. Interestingly, each of these themes derive their support from different kind of experiments. The loop structure theme is predominantly supported by light microscopy experiments [75,88-90] and the fractal theme by chromosome conformation capture [91], small angle neutron scattering [92] and tracer diffusion [93] experiments. Although the two themes are not mutually exclusive, it is often possible to classify the polymer models based on them. Here, we follow this classification in discussing the polymer models and their central features.

**Figure 5:**
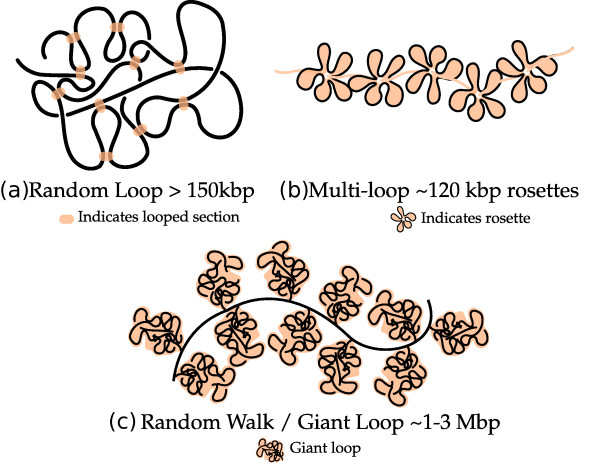
**Loop Models**. Illustration of the different loop models; (a) Random Loop model indicating loops at all scales > 150 kbp [90], (b) multi-loop subcompartment model with 120 kbp rosette structures [96] and (c) random-walk-giant-loop model with giant loops organized along a random backbone [75].

The earliest polymer model [94] considered the organization as a classical fractal where the recurring motif is that of the confined Gaussian chain. The confinement idea was introduced to explain the observation that the geometric separation between two points in the structure follows a Gaussian polymer model between the 0.1-1.5 Mbp genomic separation scale [88] while beyond this scale the geometric separation tends to become independent of genomic separation. However, an alternative proposition explaining this leveling off of geometric separation through incorporation of loops was found to be consistent with the observation of loop structures in different light microscopy experiments [95]. The loop proposition consequently led to the formulation of the random walk giant loop (RW-GL) model [75] followed by increasingly refined models like the multiloop subcompartment (MLS) [96] and the random loop (RL) [97] (Figure 5).

**Figure 6:**
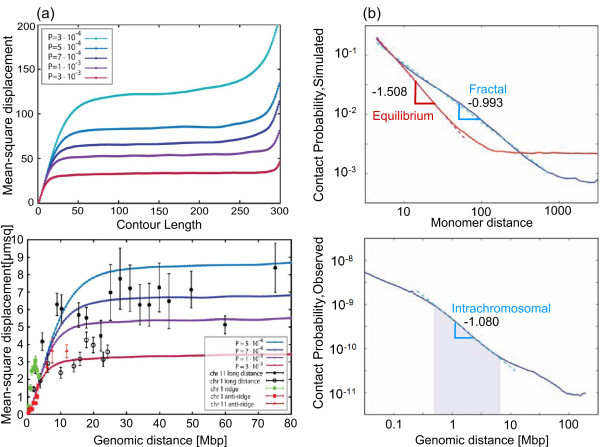
**Random Loop and Fractal Globule**. (a) Predictions of the random loop model for mean-square displacement (adapted from Mateos-Langerak et al [90]). (b) Predictions of the fractal globule model for probability of contact (adapted from the article of Lieberman-Aiden et al with permission from AAAS [91]).

The basic feature of the RW-GL model is the existence of 1-3 Mbp size loops along a randomly oriented backbone [75]. This analytical model explained the observed leveling off of 2D mean geometric distance in the 10-200 Mbp genomic distance range fairly well. However, it had limitations in capturing the compartmentalized chromosome territory structure and spatial chromatin distribution observed in experiments [96]. These limitations were attributed to the use of phantom chains in the model [96]. The MLS model based simulations, with excluded-volume interactions and subcompartments consisting of 120 kbp loops that can be opened up to accommodate giant loops, overcame the limitations of the RW-GL model and correctly predicted the 2D mean geometric distances [96]. The RW-GL and the MLS models demonstrated beyond doubt the usefulness of simulations based on polymer models for study of higher-order structures of the genome. However, these models were unable to explain the drastic leveling off of 3D geometric distances above the 10 Mbp range that were observed by recent 3D-FISH experiments [90]. The random loop model that proposed existence of loops at all scales *>*150 kbp could successfully explain this leveling off beyond the 10 Mbp scale (Figure 6). A key feature of this model is that it accounts for observed experimental features of existence of loops at several scales along with a means to account for cell-to-cell variation of measurements through averaging over different configurations of loops [97]. Further, the presence of random loops is expected to lead to formation of segregated chromosome territories as they repel each other more strongly than the linear structures [98].

The proposed loop models show a power-law behavior for geometric distances versus contour length at scales <10 Mbp, with scaling exponents ranging from *ν *= 1/3-1/2. This suggests two different internal structures at work: (1) equilibrium/globular state at short scales and (2) random looped structure at large scales. The transition between these structures is not clearly understood. In contrast, the confined fractals do not encounter these difficulties as they propose a single size exponent at all scales. Recent advances in chromosome conformation capture [91] have enabled genome-wide measurements of probability of formation of loops as a function of the size of the loops in the human genome (Figure 6). Comparison of the probability measurements with confined freely jointed chain (FJC) simulations indicate that the human genome may be organized as a fractal globule with size scaling exponent *ν *= 1/3 [91]. However, we note that the agreement of the probability measurements is observed only in the 1-10 Mbp range (Figure 6).

**Figure 7:**
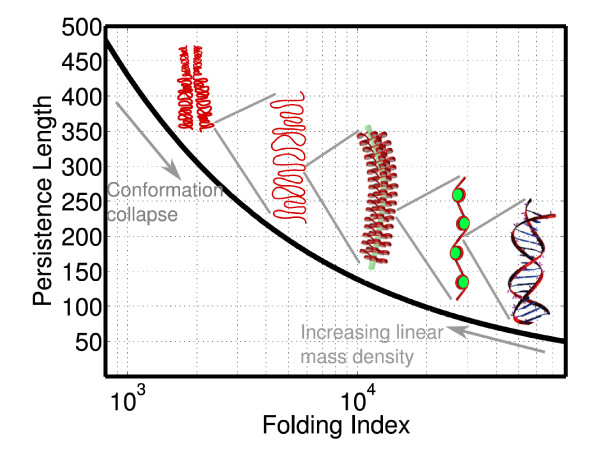
**Persistence Length Vs Folding Index**. Plot illustrating persistence length as a function of folding index for *ν *= 1/3. The folding index is analogous to the resolution of a lens through which the genome is viewed and the persistence length corresponds to the length scale of observable correlations at this specific resolution.

The advantage of this simplified picture of confined fractal is that the universal exponent *ν *observed in these experiments can be readily used to glean information on the higher-order structure by using simple ideas from the FJC model. For instance, we can define the "folding" index Φ of chromatin as the ratio of fully extended contour length *L_c _*to the folded size *C_s_*. The persistence length *L_p _*can now be computed as a function of Φ at different levels of human genome organization and is given by: Φ = (*C*_*s*,0 _/2*L*_*p*_)^2^, where *C*_*s*,0 _≈ 25 *μ*m is the size of a typical human cell nucleus (see Appendix for complete derivation) (Figure 7). Although this relationship is valid for the human genome, it has to be appropriately modified for the yeast genome, which is expected to have an organization different from that of higher eukaryotes. In particular, experiments on yeast *S. cerevisiae *indicate a linear increase in inter-chromosomal contact with the genomic size *s *of the chromosome [99]. Such an increase is expected from an equilibrium globule organization where the average size *R *of a chromosome scales as *s*^1/2 ^and hence the surface area of contact *R*^2 ^scales as *s*.

The simple arguments we used for the persistence length calculations inherently assume that the scaling exponent *ν *= 1/3 is universal within the human genome. However, simulations investigating properties of compact polymers have pointed out that the chromatin fiber, although in a state that resembles the fractal globular state of the compact polymers with *ν *= 1/3, shows deviations from these type of polymers [100]. Specifically, the moment ratios calculated from intrachain distance distributions for chromatin are observed to be different from that observed for globular state [100]. Instead, the intrachain distributions are observed to be reasonably approximated by the random loop model [100]. However, the random loop model also shows that the distances become independent of genomic separations, i.e., *ν *→ 0. The anomaly in these results can possibly be explained by the argument that in the presence of loops it is inappropriate to use the intrachain distances to determine the scaling exponent *ν*. Instead, the radius of gyration, a measure of size for closed structures like rings, may be a more appropriate quantity for determining the exponent.

We end this section with a perspective on combined use of the information at the gene locus level and the chromosomal level to infer qualitative aspects governing the functional state of the cell. In the previous section, we have shown that the decrease in persistence length *L_p _*causes a collapse and consequent *increase *in the concentration (density) of segments in a gene locus, via Eqs. (2) and (3). In contrast, in this section, we have shown using Figure 7 that a decrease in *L_p _*is accompanied by a *decrease *in the linear mass density *c *(since *c *and Φ are inversely related). The paradoxical nature of this result indicates that chromatin remodeling events, which lower the effective persistence length of chromatin, could have opposing effects on gene-related activities like transcription. On one hand, locus-wide remodeling could open up regions for transcription and lead to increased frequency of interactions between promoters and enhancers; while on the other hand, genome-wide remodeling events could cause genome folding leading to formation of inaccessible, dense regions and potential suppression of transcription. Thus, nucleosome depletion, in addition to its established role in gene activation, may also lead to repression. Such opposing effects are in fact observed when the nucleosomal content is reduced through H4 depletion in the yeast genome, resulting in increased expression in 15% and reduced expression in 10% of genes [101].

## Future Challenges

Understanding genome organization within the cell nucleus and its functional implications are fundamental issues in modern cell biology. In this article, we have reviewed the incisive role played by polymer theory and simulations in providing new insights into the structure of the genome at various levels of organization, ranging from packing of nucleosomes into the 30-nm chromatin fiber to the higher-order folding of chromatin via looping into chromosomes. Despite enormous progress in both experimental and computational fronts, a comprehensive model of genome organization at the chromatin and chromosome level is still lacking. Below we list four main challenges that computational models would have to overcome to tackle this highly intriguing and important problem.

First, genome organization is heterogeneous. At the chromatin level, this heterogeneity arises from variations in the nucleosome repeat length, nucleosome composition (histone modifications, histone variants, and linker histones), and potential entrapment of chromatin in metastable states. Most models of chromatin, including those developed in our group, tend to assume uniform nucleosome positioning and composition. Only recently have studies begun to examine the effects of chromatin heterogeneity; preliminary work in this direction has already yielded some very interesting results. At the chromosome level, the size, shape and location of chromatin loops is not fixed. Thus, a key aspect towards examining heterogeneity in chromosome models would be through more realistic modeling of the interactions across chromatin fibers, e.g., allowing loops to dynamically form and break according to the associated energetics of loop formation in chromosome models, as opposed to "fixing" looping points and loop sizes.

Second, the chromatin fiber and chromosomes are highly dynamic. Nucleosomes are constantly being displaced, modified, dissolved and reformed through various mechanisms. At the higher scale, chromatin loops are continuously being broken and reformed. In addition, a range of nuclear proteins including transcription factors and architectural proteins dynamically bind and dissociate from chromatin [2]. Such binding/dissociation events could affect chromatin structure and in turn may be affected by chromatin structure. Most models currently look at the genome from a static perspective. Incorporation of the above mentioned kinetic features into polymer models coupled with structural heterogeneity would be a crucial step in building more comprehensive models of the genome.

Third, development of accurate, coarse-grained models of chromosomes poses a grand challenge. One potential route is via "multi-scale" approaches, where each level represents coarse-graining of the previous, higher-resolution level. Such an approach generally involves the use of potential of mean forces (PMFs) for treating the interactions between coarse-grained subunits. The PMFs account for effects of the degrees of freedom that are "averaged out" during each coarse-graining step. The challenge is to maintain self-consistency from one level to another, as the PMFs are valid only when there is a clear separation of length and time scales across the levels [102]. When there is no clear separation of time/length scales, one might need to resort to "multi-resolution" approaches. In this approach, different portions of the system are treated at different resolutions, depending on their relative importance to the phenomena being investigated (in the same spirit as the QM/MM method [103] employed in enzyme catalysis). The challenge here is to identify the portions of the system (or degrees of freedom) important enough to be treated at higher resolution as opposed to those regions that can be treated at lower resolution. Another challenge is developing suitable potentials for linking low- and high-resolution regions.

Fourth, little modeling effort has been invested in determining structure-function relationships in genome organization. A definitive connection between structure and function comes from the observation that gene density is high in largely decondensed euchromatin and low in highly condensed heterochromatin [104]. Further, gene-rich and gene-poor regions are found to be physically separated from each other [105,106]. Specifically, in human lymphocytes gene-rich regions are positioned in the nuclear interior while the gene-poor regions are positioned towards its periphery [107]. Apart from gene density patterns, the molecular species responsible for nuclear processes and gene expression are connected through a complex and extensively coupled network giving rise to functional coupling between macromolecular structures and compartments [108,109]. Though many aspects of these studies are been driven by the experiments, polymer theory and simulations could contribute to better understanding of such structure-function relationships.

## Appendix

### Effect of persistence length modifications on gene locus conformation

To study the generic effects of changes in the persistence length of the chromatin fiber on the conformation of the gene locus, we have treated the chromatin fiber as a worm-like chain (WLC) with fixed contour length *L_c_*. The WLC is the standard model used to describe semi-flexible polymer chains, and it has been successfully used to explain the conformational properties of double-stranded DNA and other biopolymers [110]. Though this model is too idealized to represent *in vivo *chromatin (as it neglects fiber-fiber interactions, existence of stable loops and heterogeneous flexibility), it can nonetheless provide qualitative insights into the magnitude of the conformational changes.

We begin by writing down the mean squared end-to-end distance of a WLC with an "effective" persistence length *L_p _*of the chromatin fiber that embodies all effects of the remodeling [111]:(1)

We consider *L_p _*as a variable that changes from an initial value  to a final value  due to chromatin remodeling. Because we anticipate a weak effect of remodeling on the overall contour length at the locus level, we hold *L_c _*constant. In the ideal chain limit of the WLC, *L_c _*≫ *L_p_*, the ratio of the final to initial mean square end-to-end distances is given by(2)

Plugging in values of initial and final *L_p _*observed by Heermann and coworkers [78], the ratio becomes 140/280 = 1/2. We have further confirmed this conformational collapse by using simple lattice MC simulations of phantom linear chains of different lengths and regions of flexibility. The simulations indicate that introduction of regions of flexibility in an overall stiff chain, differing by a nominal bending energy of 2*k_B_T *per segment, strongly decreases the mean squared radius of gyration of the chains (Table 1).

**Table 1:** Ratio of mean-square radius of gyration of chains

*N_r_*	*F^a^*	*R*(0)*^b^*	*R*(1)	*R*(2)	*R*(3)
16	5.15	2.38	1.46	1.30	1.19
32	10.11	2.73	1.77	1.622	1.49
64	20.16	2.91	1.91	1.83	1.76
128	40.20	3.02	2.02	1.95	1.91
256	80.46	3.07	2.06	2.00	1.98
512	160.78	3.10	2.11	2.04	2.03
1024	321.84	3.11	2.10	2.05	2.05

Ratio of mean-square radius of gyration of chains of different lengths (*N_r_*) with different regions of flexibility to that of a fully flexible chain (F)

*^a ^*Absolute values of mean square radius of gyration

*^b ^*In R(x), x denotes the number of flexible regions sandwiched between stiff regions that are of the same length as the stiff regions.

The implications to this modification in size can be explored through the Flory approximation. According to the Flory approximation, the number of binary interactions in a polymer chain *n_b _*~ *c*^2^, where *c *is the concentration of segments [112]. Since *c *~ 1*/V *(where *V *is the volume of the chain) and  assuming spherical symmetry, the ratio of the final to the initial number of binary interactions is given by:(3)

In the case of the *IgH *locus, this translates to an 8-fold increase in *n_b_*.

### Persistence length as a function of folding index

We treat the chromatin fiber as a FJC at the genome-wide level with a contour length given by:(4)

where *N_r _*is the number of repeat units, each of length *l *= 2*L_p_*. The characteristic size of a FJC is given by [113]:(5)

where *ν *is a scaling exponent that connects the size of the chain to its number of repeat units. Note that the fractal dimension, *d_f _*, is the exponent that connects the mass of the chain *M *to its size via  and that *M *is also proportional to the number of repeat units *M *~ *N_r _*~ (*C_s_*)^1*/ν *^(from Eq. 5). Hence, *ν *is the inverse of the fractal dimension *ν *= 1*/d_f _*and determines the organization of the FJC at all length scales.

We can define the "folding" index Φ of FJC as the ratio of fully extended contour length *L_c _*to the folded size *C_s_*, which can be simplified further using Eqs. (4) and (5):(6)

Since the contour length *L_c _*= *d/c*, where *d *is the genomic distance in bp and *c *is the linear mass density in bp/nm, Φ increases with decreasing linear mass density.

The contour length within the FJC framework can be partitioned arbitrarily, i.e., if the contour length of a structure is 100 *μ*m then it can be partitioned as, say, 100 repeat units each of 1 *μ*m length or 1000 repeat units of length 0.1 *μ*m. The flexibility of the contour length partitioning allows for choice of either number of repeat units *N_r _*or the persistence length *L_p _*as a variable in Eq. (6). However, the contour length *L_c _*according to Eq. (6) is determined by the product of the folding index Φ and size *C_s_*. This constraint has to be satisfied when *L_p _*is chosen as the variable and the contour length in the framework changes with the change in the folding index. Thus Φ can be used like a lens of variable resolution to view genome organization at different length scales.

The persistence length *L_p _*can now be computed as a function of Φ at different levels of human genome organization. We begin with the organization at the level of dsDNA, whose persistence and contour lengths are known: *L_c _*≈ 2m for dsDNA with 6 billion bp at a linear mass density *c *= 2.94 bp/nm [114]. Given that *L_p _*= 50 nm [114] and *ν *= 1/3, the number of repeat units *N_r _≈ *2 *× *10^7 ^from Eq. (4), the folding index Φ *≈ *8 *× *10^4 ^from Eq. (6), and *C_s _*≈ 25 *μ*m from Eq. (5). Interestingly, the FJC size *C_s _*is consistent with typical dimensions of human cell nuclei. Since, this size is *invariant *across all organization levels, we denote it as *C*_*s*,0_. With this constraint, Eq. (6) reduces to(7)

Equation (7) thus provides the relationship between the persistence length *L_p _*and folding index Φ for all organization levels, which is plotted in Figure 7.

## Authors' contributions

BVSI and GA organized the article, contributed to the writing of all sections of the article, and developed the theoretical analysis presented in the Appendices. MK designed some of the figures and contributed to the writing of the section on secondary structure. All the authors have read and approved the final manuscript.
